# Supplementary material for: Improved Prediction Accuracy for Late-Onset Preeclampsia Using cfRNA Profiles: A Comparative Study of Marker Selection Strategies
Source: Healthcare (Basel). 2025 May 16;13(10):1162. doi: 10.3390/healthcare13101162 (PMC12110820; doi:10.3390/healthcare13101162)
Supplement: Supplementary file 1 [file healthcare-13-01162-s001.zip › healthcare-3571091-supplementary.pdf]

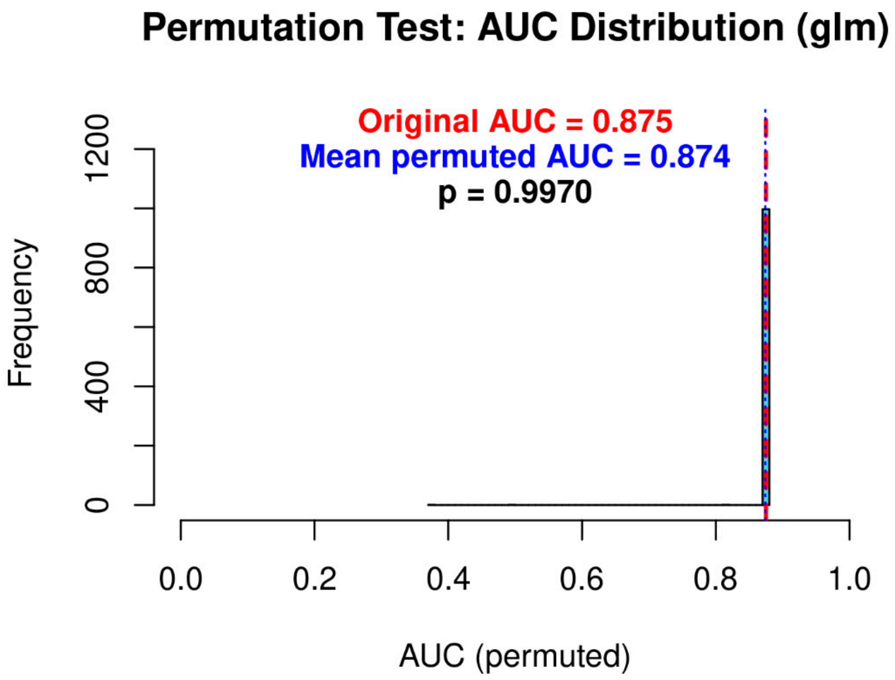

Supplementary Figure S1. Label-permutation test for the KLRC4 single-gene model.

The histogram shows the distribution of AUROC values obtained after 1 000 random shuffles of the case-control labels (*grey bars*). The vertical red dashed line marks the observed AUROC of the original logistic-regression model (0.875); the blue solid line marks the mean AUROC across permutations (0.874 ± 0.044). The one-sided empirical *p*-value (0.997) indicates that the model's discrimination is statistically indistinguishable from chance, supporting the conclusion that perfect or near-perfect performance is likely an over-fit of the small dataset.

Supplementary Table S1. Maternal demographic characteristics of the cfRNA cohort. Mean ± SD values and unpaired two-tailed t-test p-values compare each pre-eclampsia subtype with its gestational-age-matched controls. Groups did not differ in age or sampling week (all $p > 0.5$); maternal BMI was significantly higher in the LO-PE group than in its matched controls ($p = 0.036$).

	Maternal Age (years)	Maternal BMI (kg/m^2)	Gestational Age (weeks)
EO-PE Mean ± SD	29.3 ± 3.5	33.6 ± 9.0	29.2 ± 2.3
EO-Control Mean ± SD	30.1 ± 3.8	28.5 ± 7.0	29.3 ± 2.3
p-value (EO vs Control)	0.597	0.136	0.916
LO-PE Mean ± SD	30.2 ± 4.8	32.2 ± 4.9	35.6 ± 1.3
LO-Control Mean ± SD	29.4 ± 3.2	27.9 ± 4.5	35.9 ± 0.8
p-value (LO vs Control)	0.636	0.036	0.505

Supplementary Table S2. Baseline characteristics of the two normotensive control groups. Values are mean ± SD; p-values derive from two-sided Welch's t-tests except gestational age, which was predetermined by matching to the corresponding PE cases (therefore no statistical test was performed). No significant differences were detected in maternal age or BMI between EO- and LO-controls (both $p > 0.5$).

Variable	EO-Control (n = 12)	LO-Control (n = 12)	p-value*
Maternal age (years)	30.1 ± 3.8	29.4 ± 3.2	0.58
BMI ($kg\ m^{-2}$)	28.5 ± 7.0	27.9 ± 4.5	0.81
Gestational age at sampling (weeks) †	29.3 ± 2.3	35.9 ± 0.8	‡

* two-sided Welch's t-test † by design, matched to respective case groups ‡ not tested.

Supplementary Table S3. Ten-repeat stratified Monte-Carlo cross-validation performance for early-onset pre-eclampsia: mean ± SD AUROC and F1 for the proposed Elastic-Net model versus three conventional classifiers (Random Forest, linear SVM and XGBoost).

Early-onset PE (EO-PE)	AUC(mean±sd)	F1(mean±sd)
Proposed Method	0.911(0.126)	0.785(0.165)
Random Forest	0.867(0.115)	0.681(0.227)
Linear SVM	0.833(0.141)	0.751(0.166)
XGBoost	0.744(0.223)	0.705(0.188)

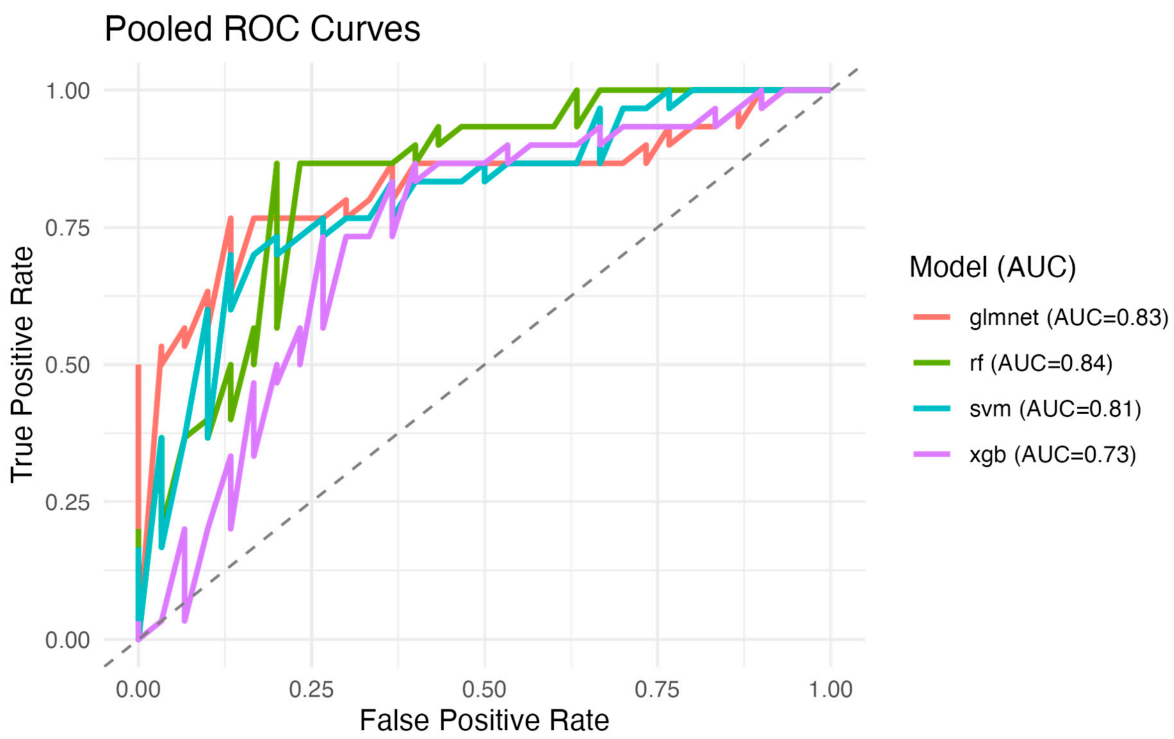

Supplementary Figure S2. Pooled ROC curves for early-onset PE. The red line (Elastic-Net) dominates the plot with the highest AUROC (~0.91), followed by green (Random Forest, ~0.87), cyan (linear SVM, ~0.83) and violet (XGBoost, ~0.74); the grey dashed diagonal denotes chance performance.

Supplementary Table S4. Ten-repeat stratified Monte-Carlo cross-validation results for late-onset pre-eclampsia: mean ± SD AUROC and F1 comparing the proposed Elastic-Net model with Random Forest, linear SVM and XGBoost baselines.

Late-onset PE (LO-PE)	AUC(mean±sd)	F1(mean±sd)
Proposed Method	0.833(0.159)	0.725(0.173)
Random Forest	0.711(0.167)	0.570(0.207)
Linear SVM	0.733(0.108)	0.532(0.149)
XGBoost	0.611(0.108)	0.615(0.231)

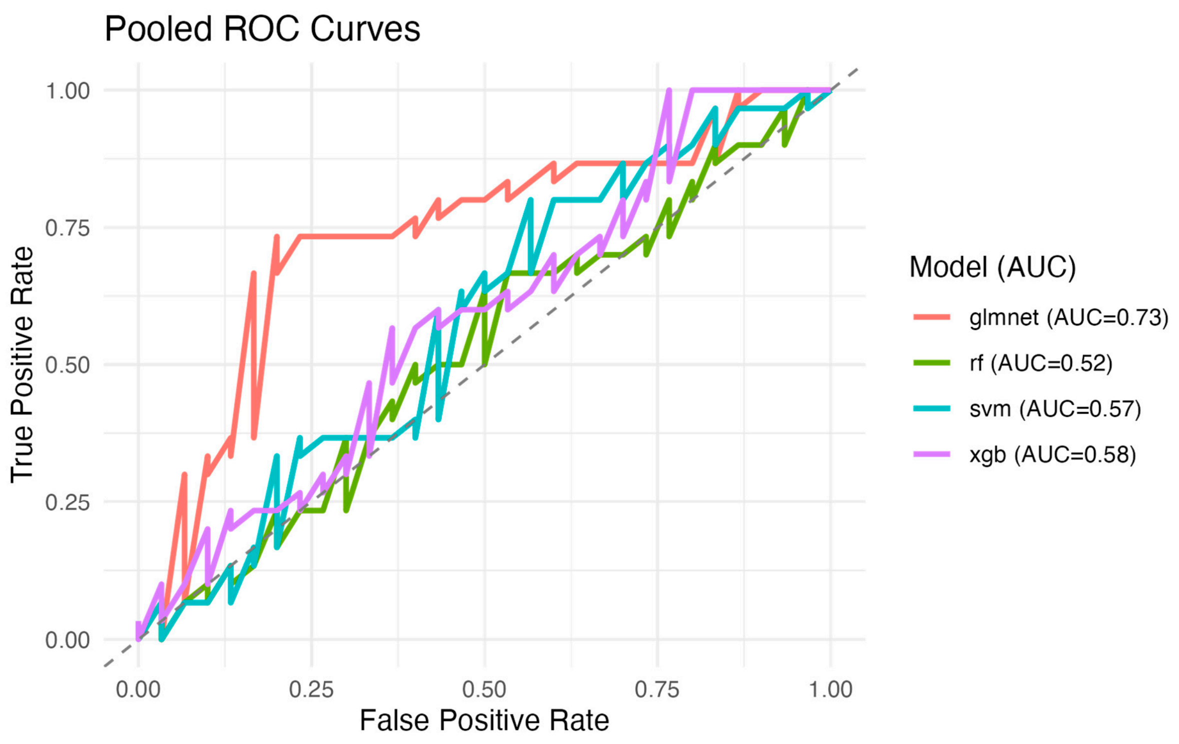

Supplementary Figure S3. pooled ROC curves for late-onset PE. Elastic-Net (red) again leads with an AUROC of ~0.83, while Random Forest (green, ~0.71), linear SVM (cyan, ~0.73) and XGBoost (violet, ~0.61) trail closer to the 45° chance line (grey dashes).
